# Development of complex I PET radiotracers

**DOI:** 10.1002/alz70856_099425

**Published:** 2025-12-25

**Authors:** Jannatun Namme, Yulong Xu, Changning Wang, Shijun Zhang

**Affiliations:** ^1^ Virginia Commonwealth University, Richmond, VA, USA; ^2^ Massachusetts General Hospital, Boston, MA, USA; ^3^ MASSACHUSETTS GENERAL HOSPITAL, Charlestown, MA, USA

## Abstract

**Background:**

Mitochondrial dysfunction has been indicated in the development of AD/ADRD. Complex I defects from the ETC of mitochondrial respiration have been associated with ROS production and neurodegeneration. Herein, novel mitochondrial complex I inhibitors were synthesized and biologically characterized to develop complex I PET radiotracers

**Method:**

Radiochemistry was conducted to radiolabel the identified complex I inhibitor with F‐18 and the radiotracer was characterized in mice with PET/CT imaging for its biodistribution and brain penetration.

**Result:**

The radiotracer can quickly enter the brain and shows a promising clearance profile. Blocking studies with the parent compound demonstrated good specificity.

**Conclusion:**

A new complex I PET radiotracer was successfully synthesized and characterized in mice by PET/CT studies.

